# Investigation on the Strengthening Mechanism of Flow Control Extrusion by Using Experiment and Numerical Simulation

**DOI:** 10.3390/ma14175001

**Published:** 2021-09-01

**Authors:** Guangshan Wu, Yangqi Li, Fei Chen

**Affiliations:** Department of Plasticity Technology, National Engineering Research Center of Die and Mold CAD, Shanghai Jiao Tong University, 1954 Huashan Road, Shanghai 200030, China; wuguangshan@sjtu.edu.cn (G.W.); liyangqi_93@sjtu.edu.cn (Y.L.)

**Keywords:** heterostructure, strengthening mechanism, flow control extrusion

## Abstract

Bimodal grain structure leads to high strength and strain hardening effect of metallic materials. In this study, an effective approach called flow control extrusion (FCE) is proposed to achieve heterostructures of pure copper. Compared with conventional extrusion (CE), FCE shows much stronger grain refine ability and much weaker grain orientation concentration. The significant grain refinement and heterostructures depend on the severe shear strain from FCE. The heterostructures of sample subject to FCE transfer from bimodal structure to gradient structure with the decrease of temperature, as the grains in the surface of sample are all refined to ultrafine scale. Both these two heterostructures can realize the improvement of strength and strain hardening effect simultaneously.

## 1. Introduction

One of the main strengthening mechanisms for polycrystalline materials is grain boundary strengthening [1,2,3,4]. The main solution to realize the grain refinement is the severe plastic deformation technique. However, it is commonly known that grain refinement strengthening also leads to the decrease of strain hardening effect and ductility [5,6]. To overcome the disadvantages, heterostructured materials have been proposed in recent years [7,8,9]. Heterostructured materials means controlling the distribution of microstructures in a three-dimensional space, which has been proved to be effective in improving the strength and ductility simultaneously by enhancing the strain hardening effect [10,11,12,13]. In the current stage, heterostructured materials usually mean the combination of ultrafine/nano grain and coarse grain with regular distribution [14,15,16,17,18]. There are mainly three categories: bimodal structure with micrometre-sized grains and ultrafine/nano grains distributes in the sample uniformly [19]; gradient structure characterized by the grain size from nano scale at the surface to micro scale in the core; and heterogeneous lamella structure with the soft micrograined lamellae embedded in hard ultrafine-grained lamella matrix [20,21,22,23]. However, obtaining the heterostructured materials is relatively complicated, which hinders the application of these strategies.

Some researchers tried to obtain heterostructures materials by simple deformation, such as vortex extrusion (VE) or twist extrusion (TE), but the advantages of heterostructured materials have not been investigated in the processed sample owing to the limited grain refinement ability [24,25,26]. On the other side, plastic deformation always leads to the generation of texture. Owing to the intrinsic plastic anisotropy of grains, the formed texture has significant effect on the mechanical properties of polycrystals [27,28,29,30]. If the metal has a strong texture, it means the anisotropy of the mechanical properties, and finally limits the following deformation strategies, particularly for scaling-up to industrial size samples [31,32,33,34]. As texture has an important effect on the properties and applications, controlling the texture is critical in practice. For the traditional deformation process (including severe plastic deformation, such as equal channel angle pressing (ECAP) and high pressure torsion (HPT), the texture is relatively strong, as the metal flow direction is constant and unified [35,36]. Different deformation processes always attribute to distinct texture components as the metal flow direction is different [37,38,39,40,41,42,43]. Hence, controlling of the metal flow plays a key role in controlling the texture of the formed parts.

In this study, a new processing technology was proposed to achieve a significant grain refinement and meanwhile prevent the concentrated grain orientation with a single metal flow pattern. The proposed technology is quite different from those traditional deformation modes based on the characteristic that the grain orientations distribute along the deformation direction. Meanwhile, the developed processing strategy also results in the formation of the heterostructures and improves the strength and strain hardening effect simultaneously.

## 2. Experiments

### 2.1. Principle of Flow Control Extrusion (FCE)

**Figure 1:**
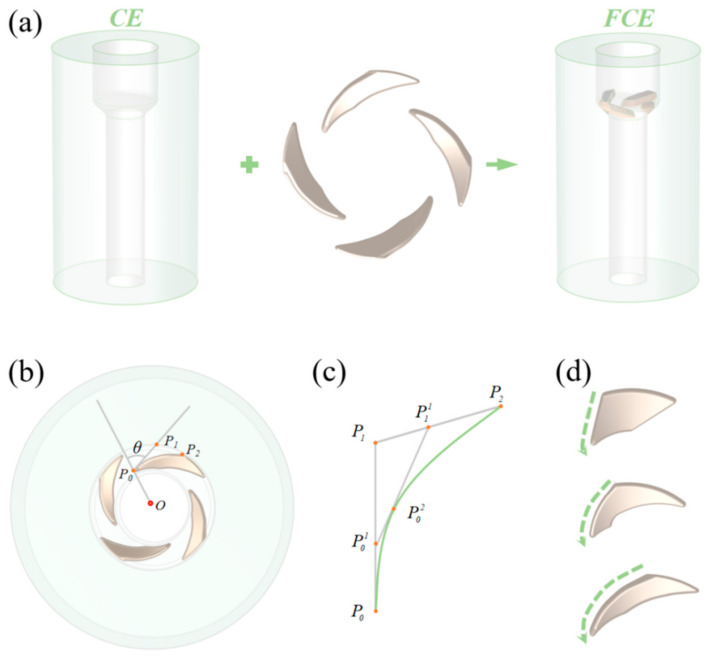
Schematic illustration of flow control extrusion (FCE): (**a**) the principle of FCE, (**b**) the control of bulges, *P*_0_, *P*_1_, and *P*_2_ are points located at the edge of deformation zone; (**c**) the structure design of bulges, *P*_0_^1^ and *P*_1_^1^ mean the moving point on *P*_0_*P*_1_ and *P*_1_*P*_2_ respectively, and the trajectory of *P*_0_^2^ constructs the curve *P*_0_*P*_2_; (**d**) severe structure of bulges with increase radian.

Figure 1a shows the schematic of FCE, which has several bulges in the deformation zone of extrusion die. The flow direction and path of the sample can be controlled by the designed structure and distribution of these bulges, and thus affect the grain refinement ability and grain orientations. As shown in Figure 1b, the distribution of the bulges is controlled by θ, which represents the angle between O*P*_0_ and *P*_0_*P*_1_. The range is from 0° to 90°. *P*_0_, *P*_1,_ and *P*_2_ are located at the edge of deformation zone. To be precise, *P*_0_ is the intersection point of inner circle and the edge of bulge. *P*_0_*P*_1_ represents the tangent of edge through *P*_0_. *P*_1_ represents the intersection point of the tangent and outer circle. O presents the center of deformation areas. *P*_2_ represents the intersection of outer circle and the edge of the bulge, which can be changed to alter the structure of the bulge. The profile of the bulge is a Bézier curve as defined in Equation (1), in which point $P_{0}^{1}$ and $P_{1}^{1}$ mean the moving point on *P*_0_*P*_1_ and *P*_1_*P*_2_, respectively. The trajectory of $P_{0}^{2}$ constructs the curve, when $P_{0}^{1}$ moves from *P*_0_ to *P*_1_. The angle of the bulges increases with the length of *P*_1_*P*_2,_ as shown in Figure 1c,d, which shows the severe structure of bulges with the angle increasing from top to bottom. The upper one shows the exceptional case that *P*_2_ coincides with *P*_1_. In this study, four bulges are spirally and symmetrically distributed, θ is 70°, the length of *P*_0_*P*_1_ is same with *P*_1_*P*_2_, and the height of bulges is 2 mm. It is expected that by adjusting these parameter, more precise control of grain size and orientation can be realized by FCE.

(1) $$\frac{P_{0}P_{0}^{1}}{P_{0}^{1}P_{1}}=\frac{P_{1}P_{1}^{1}}{P_{1}^{1}P_{2}}=\frac{P_{0}^{1}P_{0}^{2}}{P_{0}^{2}P_{1}^{1}}.$$

**Figure 2:**
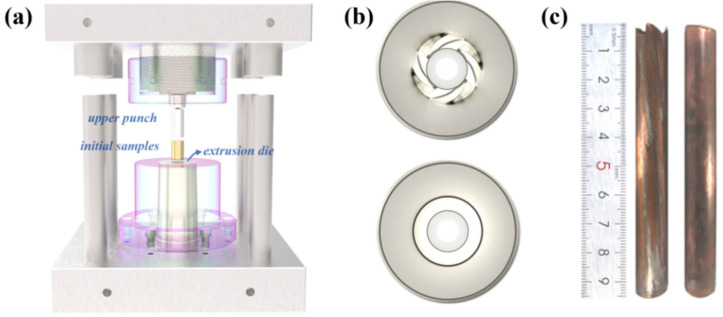
(**a**) Assembled die for FCE and conventional extrusion (CE), (**b**) the extrusion channel of FCE (**upper**) and CE (**bottom**), and (**c**) the sample after FCE (**left**) and CE (**right**).

Cylindrical billets of annealed (390 °C, 4 h) pure copper (99.99%, Huahu iron and Steel Group Co., Ltd, Shanghai, China), 30 mm in diameter and 40 mm in length, were used for the experiment. The annealed samples were formed by using FCE at room temperature and 150 °C with a pressing velocity of 3 mm/s. Conventional extrusion (CE) was also applied on the samples at same conditions of temperature for comparation. The experiment of FCE and CE were conduct by 320t double acting hydraulic press (Huzhou machine tool factory, Huzhou, China). The extrusion ratio of FCE and CE is 25:9. The assembled die for FCE and CE can be found in Figure 2a, in which the extrusion die is replaceable. Figure 2b shows the extrusion channel of FCE and CE die in this study, and the processed sample after FCE and CE can be found in Figure 2c.

### 2.2. Microstructure Characterization and Mechanical Property Test

**Figure 3:**
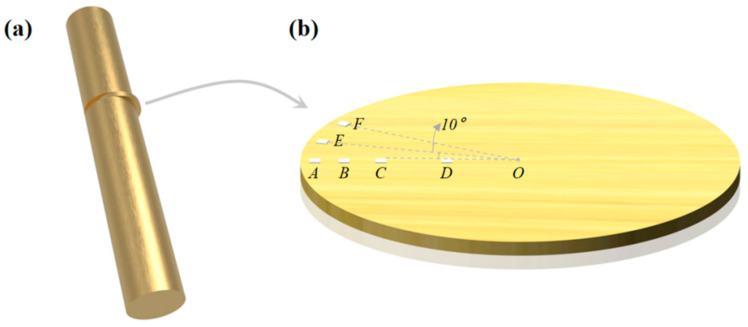
Schematic illustration of the electron backscatter diffraction (EBSD) investigated locations. (**a**) The EBSD samples were cut from the processed specimen perpendicularly to the central axis, (**b**) A–F shows the position for EBSD, where B, C, D are 1 mm, 2 mm, and 4 mm away from the edge, A, E, F are 250 μm away from the edge.

**Figure 4:**
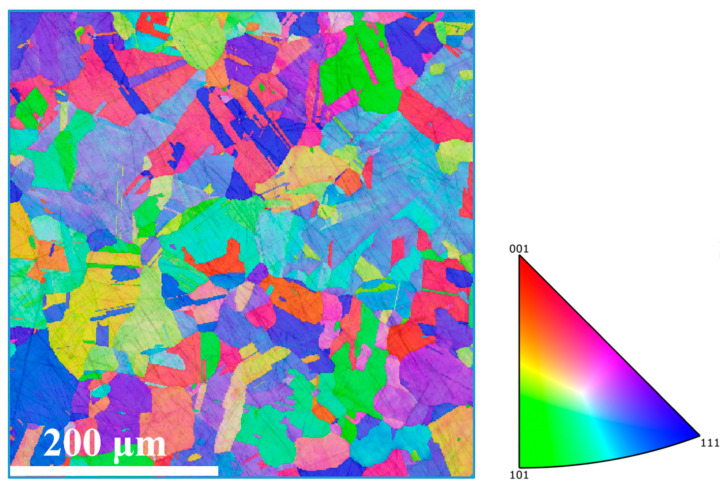
The EBSD inverse pole figure (IPF) of initial sample.

**Figure 5:**
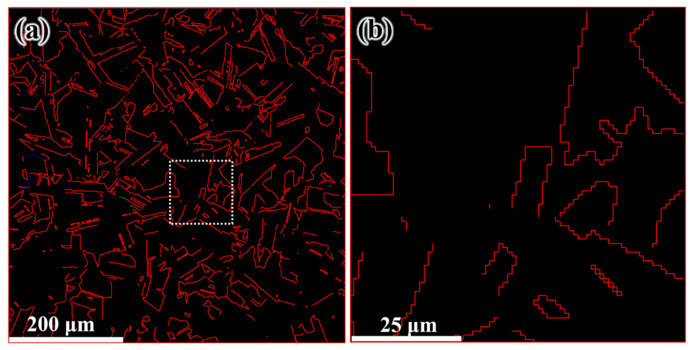
(**a**) The EBSD twin boundary map of initial sample, Σ3, Σ5, Σ7 represent different type of twin boundary and (**b**) zoomed-in image of the region within the box in (**a**).

The grain structure and texture feature of the processed specimens were examined by electron backscatter diffraction (EBSD, FEI, Hillsboro, OR, USA). The EBSD samples were cut from the processed specimen perpendicularly to the central axis, as shown in Figure 3a. After mounting, samples were ground using 600, 800, 1200, and 4000 SiC papers. Then, the diamond polishing solutions of 3, 1 and 0.3 μm were employed for further polishing. Finally, the vibration polishing (Buehler, Lake Bluff, IL, USA) was used for EBSD investigations. The four investigated areas were marked by A, B, C, and D. Their positions away from the edge of the processed sample are 250 μm, 1 mm, 2 mm, and 4 mm, respectively, as shown in Figure 3b. The positions of E and F are 250 μm away from the edge. The angle of A-O-E and E-O-F are about 10°. The processed specimen was cut vertical to its axis with a height of 16 mm and diameter of 11 mm for the compression tests. Room temperature compression tests were carried out on these specimens at a strain rate of 5.0 × 10^−3^·s^−1^. The grain size and specific surface area of grain boundary are determined by the line coincidence method. Figure 4 shows the microstructure of the initial sample, which is characterized by coarse equiaxed grains with an average size of about 60 μm. Figure 5a shows the twin boundary of the initial sample, which has a twin length per unit area of 0.026 μm^−1^. For better comparison with the processed samples, Figure 5b shows the local enlarged image of Figure 5a.

## 3. Experimental Results

### 3.1. Microstructure

**Figure 6:**
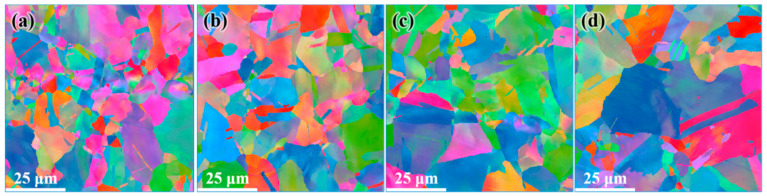
Distributions of microstructure after CE at 150 °C: (**a**) 250 μm from edge, (**b**) 1 mm from edge, (**c**) 2 mm from edge, and (**d**) 4 mm from edge.

**Figure 7:**
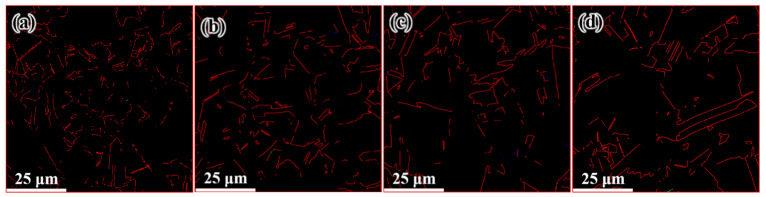
Distributions of twin boundary after CE at 150 °C: (**a**) 250 μm from edge, (**b**) 1 mm from edge, (**c**) 2 mm from edge, and (**d**) 4 mm from edge.

**Figure 8:**
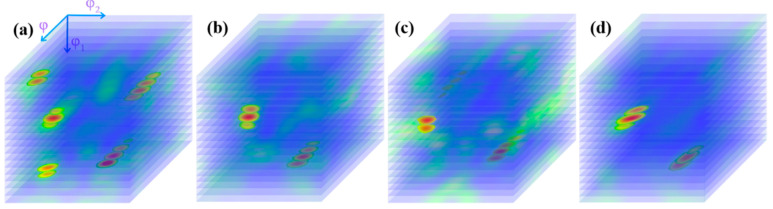
Orientation distribution function (ODF) of CEed sample at the processing temperature of 150 °C: (**a**) 250 μm from edge, (**b**) 1 mm from edge, (**c**) 2 mm from edge, and (**d**) 4 mm from edge.

Figure 6 shows the microstructure distribution of CEed sample from edge to center at a processing temperature of 150 °C. As shown in this figure, the grains have been refined effectively. The average diameter of grains near the edge was obviously reduced to 5.54 μm, and the specific surface area of grain boundaries increased to 0.36 μm^−1^. The grain refinement ability in the center is relative weak, as the average grain diameter increases to 13.4 μm and the specific surface area of grain boundaries decreases to 0.15 μm^−1^, as shown in Table 1. Furthermore, it can be seen in Figure 7 that the sample after CE at 150 °C contains more twin boundaries than the initial sample. A similar trend is observed where the twin length per unit area near the edge is higher than that in the center. The orientation distribution function (ODF) of the corresponding region is shown in Figure 8. It can be seen from the diagram that the grain orientation in the region near the edge is relatively dispersed, and the grain orientation distribution is more concentrated near the center. In general, the grain orientation of different regions is relatively consistent, as the main texture components in different regions of CEed sample are all {1 −1 1} <1 2 1> and {1 1 −1} <2 2 5>. As copper belongs to orthorhombic systems, the calculation of texture components is based on Equations (2) and (3):

(2) $$u:v:w=(\cos\varphi_{1}\cos\varphi_{2}-\sin\varphi_{1}\sin\varphi_{2}\cos\varphi ):(-\cos\varphi_{1}\sin\varphi_{2}-\sin\varphi_{1}\cos\varphi_{2}\cos\varphi ):\sin\varphi_{1}\sin\varphi$$

(3) $$h:k:l=\sin\phi \sin\phi_{2}:\sin\phi \cos\phi_{2}:\cos\phi$$

**Figure 9:**
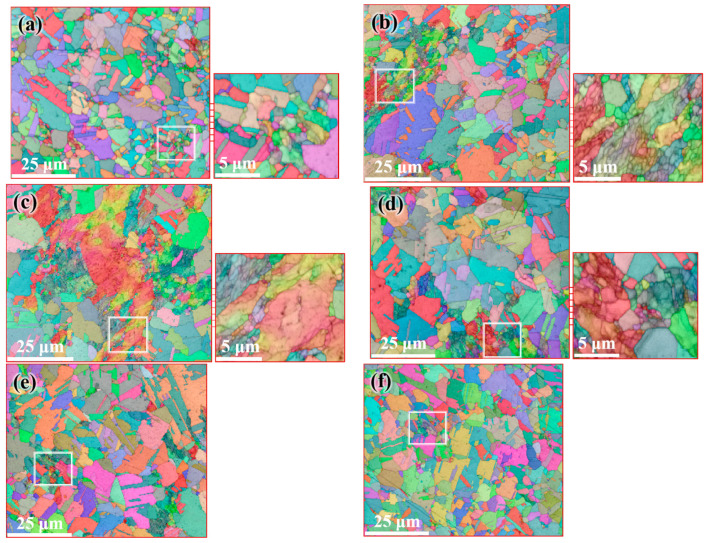
Distribution of microstructure after FCE at 150 °C: (**a**) 250 μm from the edge, (**b**) 1 mm from the edge, (**c**) 2 mm from the edge, (**d**) 4 mm from the edge, and (**e**,**f**) 250 μm from the edge.

**Figure 10:**
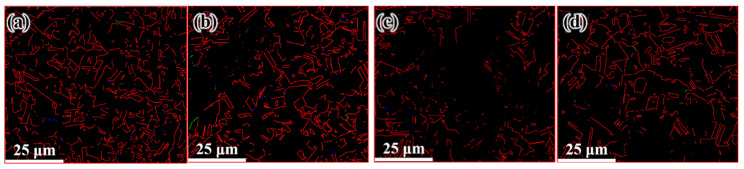
Distribution of twin boundary after FCE at 150 °C: (**a**) 250 μm from the edge, (**b**) 1 mm from the edge, (**c**) 2 mm from the edge, (**d**) 4 mm from the edge.

**Figure 11:**
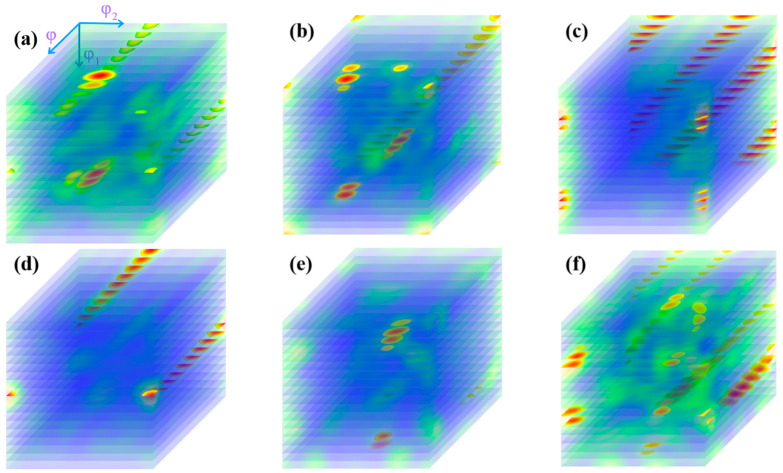
ODF of FCEed sample at the processing temperature of 150 °C: (**a**) 250 μm from the edge, (**b**) 1 mm from the edge, (**c**) 2 mm from the edge, (**d**) 4 mm from the edge and (**e**,**f**) 250 μm from the edge.

Figure 9 shows the microstructure distribution of the FCEed sample at a processing temperature of 150 °C. It can be seen that the grain refinement effect of FCE is more obvious compared with that of CE. Most grains are refined to several micron sizes, and some grains are refined to ultrafine/nano grain. Figure 9a–d shows the microstructure distribution from edge to center, which shows that the grain refinement in the edge region is more obvious than that in the center region. The test positions of Figure 9e,f are 250 μm from the edge. It is obviously found that ultrafine/nano grains exist in the whole sample. The average grain diameter increases from 2.58 μm in the edge to 3.34 μm in the center, and the specific surface area of grain boundaries decreases from 0.78 μm^−1^ in the edge to 0.60 μm^−1^ in the center, as shown in Table 2. It can be seen from Figure 10 that the twin boundaries in the area with ultrafine/nano grain are significantly less than that in the area with coarse grain, this is because most twin boundaries in the area with ultrafine/nano grain were consumed during recrystallization. Figure 11 shows the ODF in different regions of the FCEed sample at the processing temperature of 150 °C. The ODF of the whole FCEed sample is relatively dispersed and shows a distinct difference with each other. Figure 11a,e,f shows that the grain orientation in these regions is obviously quite different even their distance from the edge is identical. This is because the bulges in the reduction zone of the extrusion die lead to different flow trajectories in different regions of sample. Generally speaking, the microstructure of the FCEed sample at 150 °C is characterized by the bimodal microstructure of coarse grains combined with ultrafine/nano grains.

**Figure 12:**
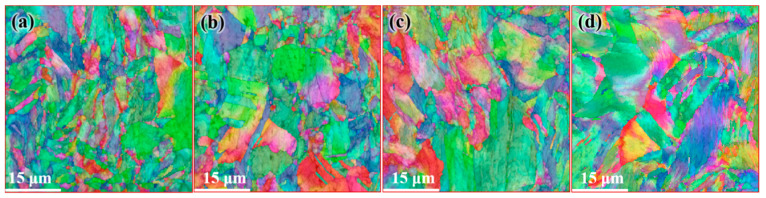
Distribution of microstructure after CE at room temperature: (**a**) 250 μm from the edge, (**b**) 1 mm from the edge, (**c**) 2 mm from the edge and (**d**) 3 mm from the edge.

**Figure 13:**
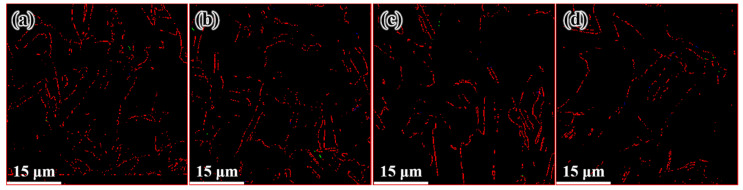
Distribution of twin boundary after CE at room temperature: (**a**) 250 μm from the edge, (**b**) 1 mm from the edge, (**c**) 2 mm from the edge and (**d**) 3 mm from the edge.

**Figure 14:**
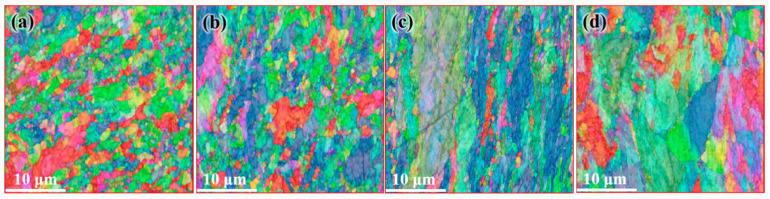
Distribution of microstructure after FCE at room temperature: (**a**) 250 μm from the edge, (**b**) 1 mm from the edge, (**c**) 2 mm from the edge, and (**d**) 3 mm from the edge.

**Figure 15:**
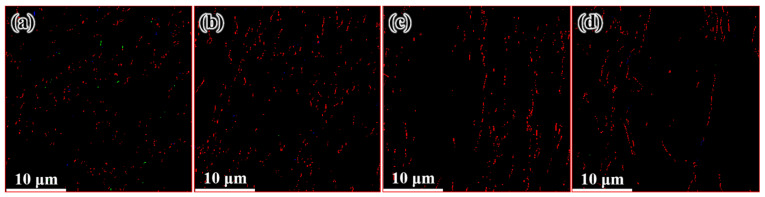
Distribution of twin boundary after FCE at room temperature: (**a**) 250 μm from the edge, (**b**) 1 mm from the edge, (**c**) 2 mm from the edge, and (**d**) 3 mm from the edge.

Figure 12 shows the distribution of the microstructure after CE at room temperature, the characterized positions are more concentrated than that of the FCEed sample, as the microstructure of sample subject to FCE at room temperature is characterized with obvious gradients. It can be seen from the diagram that grain refinement effect of the sample has been strengthened compared with that of CEed sample at 150°. The average grain diameter increases from 2.65 μm in the edge to 10.18 μm in the center, and the specific surface area of grain boundaries decreases from 0.75 μm^−1^ in the edge to 0.19 μm^−1^ in the center, as shown in Table 3. The twin boundaries of CEed sample at different processed temperatures do not show obvious difference, as shown in Figure 13 and Table 3. Figure 14 shows the microstructure and morphology distributions of the sample after FCE at room temperature. It can be seen that the thickness of the formed ultrafine/nano grain lamella is more than 1 mm. Beside the ultrafine grain lamella, the microstructure is characterized by a heterogeneous lamella structure with microcrystalline lamellae and UFG lamellae. As for the center region, the heterogeneous lamellae structure is replaced by the bimodal grain structure composed of equiaxed micron-sized grains and ultrafine grains. It can be seen from Table 4 that the average grain diameter increases from 0.72 μm in the edge to 1.47 μm in the center, and the specific surface area of grain boundaries decreases from 2.78 μm^−1^ in the edge to 1.36 μm^−1^ in the center. On the other side, because of the consumption of twins due to recrystallization, the twin boundaries of FCEed sample at room temperature are obviously less than that of samples subjected to other processing conditions, as shown in Figure 15 and Table 4.

### 3.2. Mechanical Properties

**Figure 16:**
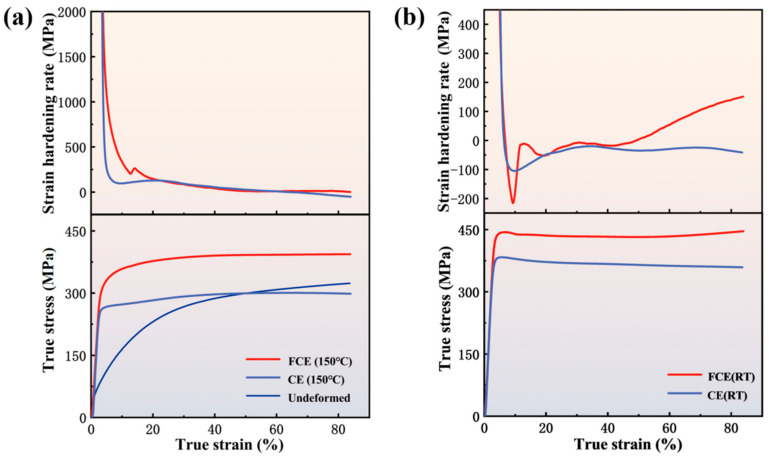
Mechanical properties of unprocessed materials, materials after CE and FCE at room temperature and 150 °C: (**a**) true stress-strain and strain hardening rate curve of unprocessed materials, materials after CE and FCE at 150 °C; (**b**) true stress-strain and strain hardening rate curve of materials after CE and FCE at room temperature.

The mechanical properties of the specimen before and after processing were characterized by compression tests at room temperature, as shown in Figure 16. The strength of the specimen after processing is obviously improved. The strength of FCEed sample is significantly higher than that of the CEed at different processing temperatures. For the sample processed at 150 °C, the yield strength of FCEed sample is 332 MPa, which is nearly 40% higher than that of the CEed sample (245 MPa). When processed at room temperature, the yield strength of FCEed sample reaches 443 MPa, while the CEed sample is 382 MPa. Meanwhile, the FCEed samples show higher work hardening rate than that of the CEed samples, but there are some differences between the FCEed sample at room temperature and the one at 150 °C. After yielding, FCEed sample at 150 °C shows a significantly higher working hardening rate than that of the CEed sample before the strain reaches 20%, and keeps relatively consistent when the strain is higher than 20%. As for the FCEed sample at room temperature, the work hardening rate shows a transient decrease after yielding, but then increases rapidly. On the other hand, both the CEed and FCEed sample at room temperature shows post-yield softening, this can be attributed to two reasons. The first one is that the samples after processing at room temperature contain relatively high dislocation density, which means some of these dislocations will annihilate during plastic deformation and lead to the work softening [44,45]. The second one is, when the grain size reduce to ultrafine scale the dislocations often glide across the grain and then disappear at GBs without accumulation in grain interior [7].

## 4. Discussions

### 4.1. Microstructure Evolution

**Figure 17:**
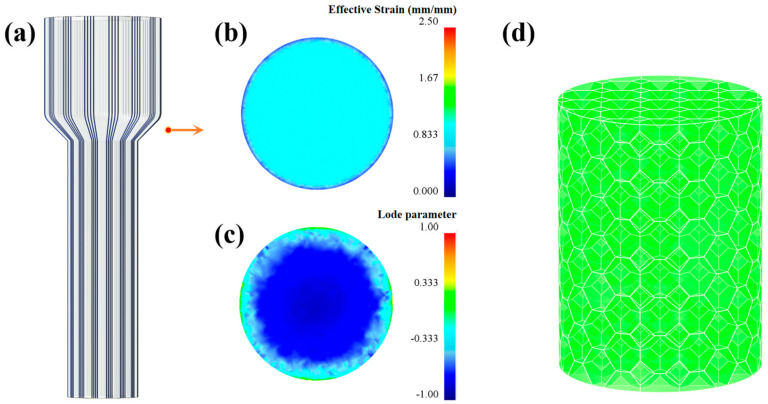
Schematic diagram summarizing the mechanism of microstructure evolution during CE: (**a**) schematic diagram of metal flow direction subject to CE; (**b**) effective strain in the deformation zone; (**c**) load parameter in the deformation zone, and (**d**) schematic diagram of the microstructure after CE.

**Figure 18:**
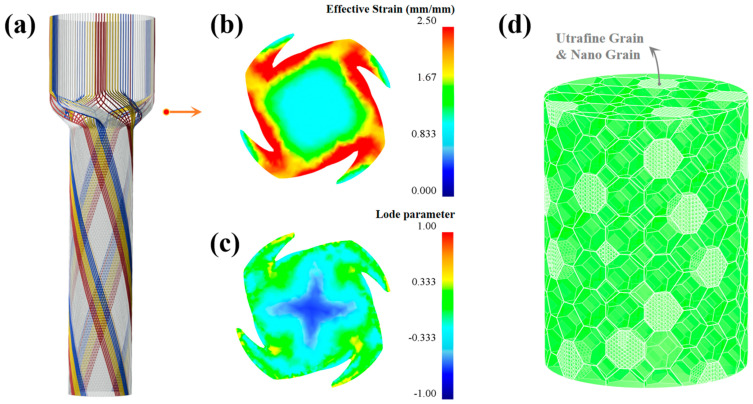
Schematic diagram summarizing the mechanism of microstructure evolution during FCE: (**a**) schematic diagram of the metal flow direction subject to FCE; (**b**) effective strain in the deformation zone; (**c**) load parameter in the deformation zone and (**d**) schematic diagram of the microstructure after FCE.

To investigate why FCE shows much more strong grain refinement ability than that of CE, a finite element model was used to study the stress and strain state during processing. The simulation was conducted using the commercial software DEFORM-3D (version 11, 2016, Scientific Forming Technologies Corporation, Columbus, OH, USA), Lode parameter (μ_σ_) was controlled by adding a user’s subroutine. Lode parameter was defined by Equation (4), where σ_1_, σ_2_ and σ_3_ are principal stress components. As shown in Figure 17, during the CE processing, the metal goes through two shear deformation stages in the turning of CEed die, while the strain is relatively small. Furthermore, it can be seen from Figure 17c that the lode parameter is close to 0 at the surface while most of the region is nearly −1, which means that the shear strain plays the key role in the surface while the tensile strain plays the key role in the other regions. Consequently, the CEed sample shows limited grain refinement ability as the tensile strain has limited effect in grain refinement compared to the shear strain. As for the FCE, the metal flows along the outline of the bulges in the reduction zone, which leads to a large strain to the metal and promotes grain refinement, as shown in Figure 18a,b. Furthermore, it can be seen from Figure 18c that the lode parameter of the sample during FCE deformation is closer to 0, which means that the shear strain plays the dominant role in FCE. As compared to tensile strain, the shear strain can promote grain refinement and the formation of twin boundaries effectively [46]. In other words, FCE is able to provide much stronger grain refinement ability and improve the generation of twin boundary, hence the strength of FCEed sample can be improved significantly. As shown in Figure 17d and Figure 18d, compared with the homogeneous coarse grain structure of CEed sample, FCE can build a bimodal structure in the sample at 150 °C. The reason is that the severe shear strain imposed by FCE accumulates in grains with a soft orientation, and leads to these grains being refined to ultrafine scale first. When the processing temperature decreases to room temperature, more severe deformation can refine all the grains in surface region to ultrafine scale, and build the gradient structure in the FCEed sample consequently. On the other hand, owing to the vectoring effect of these bulges applied on the metal flow, the materials have different flow trajectories in passing through the deformation zone. Finally, the grain orientation in different regions of the sample after FCE is quite different.

(4) $$\mu_{\sigma }=\frac{\sigma_{2}-\left(\sigma_{1}+\sigma_{3}\right)/2}{\left(\sigma_{1}-\sigma_{3}\right)/2}\;\mathrm{with}\;\sigma_{1}\geq \sigma_{2}\geq \sigma_{3}$$

### 4.2. Strengthening Mechanism

**Figure 19:**
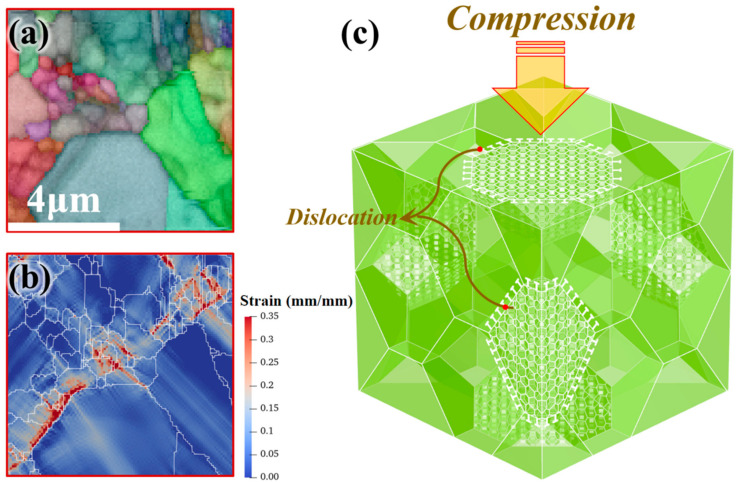
The mechanism of strain hardening effect improvement after FCE: (**a**) the microstructure for the simulated; (**b**) the simulated result by crystal plasticity spectrum method and (**c**) schematic diagram of strain strengthening rate improvement.

After yielding, the work hardening effect of FCEed samples at 150 °C is significantly stronger than that of CE. To investigate the effect of bimodal structure on the mechanical property, strain distribution of bimodal structure during compression deformation was simulated using crystal plasticity spectrum method (CPSM) by the open-source crystal plasticity simulation framework DAMASK (version 2.0.3, 2019, Max-Planck-Institut für Eisenforschung GmbH, Gottingen, Germany), the detailed theory employed in the simulation can be found in References [47,48,49], the main parameters can be seen in Table 5. Figure 19a gives the simulated microstructure. Figure 19b shows the strain distribution after compression with a strain of 10%. The result shows that the strain mainly accumulates at the boundary between ultrafine and coarse grains during the deformation. This is due to the ultrafine grain being able to hinder the movement of dislocations and cause entanglement of dislocations, while coarse grains can better produce dislocations and act as the source of dislocations, as shown in Figure 19c. Furthermore, based on the simulation results, it can be concluded that the bimodal structure formed after FCE at an elevated temperature enables the billet to accumulate dislocations quickly after yielding. However, with the accumulation of strain, the stored dislocation reaches a certain degree, and the strain hardening rate of the FCEed sample with bimodal structure approaches that of the CEed sample.

In general, heterostructure can be constructed in the processed samples using FCE at both room temperature and elevated temperature, which promotes the accumulation of dislocation and improves the strain hardening effect consequently. The strain hardening mechanism is obviously different for these two heterostructures. The possible reasons are as follows: the first reason is that the grain size of FCEed sample at room temperature is much finer than that at 150 °C, which makes the strain hardening rate reduce quickly. The second is due to the gradient structure of the FCEed sample at room temperature. Here, the sample is characterized by an ultrafine-grained structure in the surface layer, heterogeneous lamella structure inward, and gradually transits to bimodal grain structure. The strength difference among these layers induces the strain gradients and results in the long-range back stress, which is the main reason for the quick increase after decrease of the strain hardening rate [7,8,9,15,16]. The back stress increased with plastic strain contributes to the strain hardening. General speaking, the sample subjected to FCE at elevated high temperature shows a higher stain hardening rate than that at room temperature. This is because the grain refinement of sample subject to FCE at room temperature is more severe than that at an elevated high temperature, which weakens the accumulation of dislocation.

## 5. Conclusions

Bimodal structured pure copper with enhanced strength and strain hardening effect was established after FCE. The microstructure, grain orientation concentration, and mechanical properties of the sample subject to FCE were investigated in this work. The following conclusions can be drawn:

1. The bimodal structure can be realized using FCE at 150 °C, which is characterized with ultrafine/nano grains embed in coarse grains. This structure transforms to the gradient structure when the processing is conducted at room temperature, with the grains in edge region all refined to an ultrafine scale.
2. Grain orientation concentration of sample subject to FCE is much weaker than that of CE. This is because the bulges in FCE result in much more complex flow trajectories of metal.
3. The sample subject to FCE shows significant improvement of the strain hardening effect and strength compared to CE because the heterostructures were formed after FCE, while overall refinement may reduce the strain hardening effect.

## Figures and Tables

**Table 1:** The average grain diameter, specific surface area of grain boundaries, and twin length per unit area after CE at 150 °C.

Distance from Edge (mm)	Average Grain Diameter (μm)	Specific Surface Area of Grain Boundaries (μm^−^^1^)	Twin Length per Unit Area (μm^−^^1^)
0.25	5.54	0.36	0.083
1	7.92	0.25	0.076
2	11.05	0.18	0.067
4	13.4	0.15	0.061

**Table 2:** The average grain diameter, specific surface area of grain boundaries and twin length per unit area after FCE at 150 °C.

Distance from the Edge (mm)	Average Grain Diameter (μm)	Specific Surface Area of Grain Boundaries (μm^−^^1^)	Twin Length per Unit Area (μm^−^^1^)
0.25	2.58	0.78	0.21
1	2.84	0.70	0.16
2	3.23	0.62	0.10
4	3.34	0.60	0.13

**Table 3:** The average grain diameter, specific surface area of grain boundaries, and twin length per unit area after CE at room temperature.

Distance from the Edge (mm)	Average Grain Diameter (μm)	Specific Surface Area of Grain Boundaries (μm^−^^1^)	Twin Length per Unit Area (μm^−^^1^)
0.25	2.65	0.75	0.098
1	5.92	0.34	0.087
2	8.97	0.22	0.083
3	10.18	0.19	0.067

**Table 4:** The average grain diameter, specific surface area of grain boundaries, and twin length per unit area after FCE at room temperature.

Distance from the Edge (mm)	Average Grain Diameter (μm)	Specific Surface Area of Grain Boundaries (μm^−^^1^)	Twin Length per Unit Area (μm^−^^1^)
0.25	0.72	2.78	0.013
1	0.89	2.24	0.015
2	1.22	1.64	0.019
3	1.47	1.36	0.021

**Table 5:** Parameters in the constitutive model.

C_11_ (MPa)	C_12_ (MPa)	C_44_ (MPa)	h_0_ (MPa)	τ_0_ (MPa)	τ_s_ (MPa)	q	γ_0_
168,400	121,400	75,400	90	1	1.3	1	0.001

C_11_, C_12,_ and C_44_ represent the elastic moduli, h_0_ is the initial hardening modulus, τ_0_ is the initial yield stress, τ_s_ is the stage I stress, q is the s latent hardening parameters, and γ_0_ is the parameter of slip interaction.

## Data Availability

The data presented in this article are available within the article.
